# Physical and psychological aspects of anorexia nervosa based on duration of illness: a cross-sectional study

**DOI:** 10.1186/s13030-019-0173-0

**Published:** 2019-12-23

**Authors:** Shu Takakura, Chie Suzuyama Aso, Kenta Toda, Tomokazu Hata, Makoto Yamashita, Nobuyuki Sudo

**Affiliations:** 1 Department of Psychosomatic Medicine, Kyushu University Hospital, 3-1-1 Maidashi, higashi-ku, Fukuoka, 812-8582 Japan; 2 Department of Psychosomatic Medicine, Graduate School of Medical Sciences, Kyushu University, 3-1-1 Maidashi, Higashi-ku, Fukuoka, 812-8582 Japan

**Keywords:** Anorexia nervosa, Duration of illness, Physical feature, Psychological feature, Eating disorder inventory, Parental bonding inventory, Overprotection

## Abstract

**Background:**

We evaluated physical and psychological features of patients with anorexia nervosa (AN) who differed by duration of illness.

**Methods:**

Data were obtained from 204 female patients with AN, divided into two groups based on illness duration: short-term illness duration (less than 5 years; *n* = 118); and long-term duration (5 years or more; *n* = 86). Physical parameters were measured using blood serum testing and psychological aspects were assessed using various instruments.

**Results:**

A significantly higher proportion of restricting type AN was observed in the short-term group while the proportion of binge eating/purging type AN was higher in the long-term group. There was no difference in body mass index (BMI) between the groups. Serum total protein, albumin, potassium, chloride, and calcium in the long-term group were significantly lower than in the short-term group. Overall scores on the Eating Disorder Inventory as well as most of the subscales, except maturity fears, were higher in the long-term group than in the short-term group. The care subscale of the Parental Bonding Instrument (PBI) was lower in the long-term group than in the short-term group, while the overprotection subscale of the PBI was higher in the long-term group than in the short-term group. Results of a multiple regression analysis indicated that the overprotection subscale of the PBI was the only significant predictor of duration of illness.

**Conclusions:**

Duration of illness may be associated with physical and psychological features of AN; thus, adapting therapeutic approaches to illness duration might be necessary.

## Background

Anorexia nervosa (AN) is a disease that manifests as severe physical and psychosocial symptoms. The treatment of AN patients with a longer duration of illness is often more difficult than that of patients with a shorter illness duration [1, 2]. Previous reports have indicated that patients with a longer duration of AN often experience social maladjustment and physical problems because of persisting low body weight [3–5]. Hjern et al. [6] reported that 8.7% of patients with adolescent-onset AN had persistent psychiatric health problems and almost a quarter were dependent on the state for their main income. Recovery from AN was found to be less likely among patients with a longer duration of illness, but early interventions were more likely to be effective [7, 8]. Additionally, family therapy was less effective than individual therapy among patients with early-onset AN that lasted over 3 years [9]. Nozaki et al. reported that inpatients with a longer duration of AN manifested higher levels of hysteria and low back pain according to responses from the Minnesota Multiphasic Personality Inventory [10]. Furthermore, it has been reported that patients with a longer duration of AN show more deterioration of cardiac sympathovagal balance and renal function [11, 12]. Thus, treatments for patients with AN who have a longer duration of illness can be difficult because of the more severe physical and psychological features; thus, early intervention is preferable.

Previous research suggests that a sustained duration of illness may affect the clinical features of patients with AN. Consideration of illness duration may be necessary for designing appropriate interventions.

Therefore, we hypothesized that the clinical features of patients with a longer duration of AN would be more severe compared with patients with a shorter duration of illness. The current study aimed to multi-dimensionally define by illness duration the physical and psychological features of patients with AN, in in order to provide appropriate intervention based on illness duration.

## Methods

### Subjects

Data were obtained from 227 female Japanese first visit patients with AN who attended the Department of Psychosomatic Medicine at Kyushu University Hospital between April 1st, 2015 and March 31st, 2019. They were diagnosed with AN according to the Diagnostic and Statistical Manual of Mental Disorders, Fifth Edition. After 23 were excluded because of missing data, the remaining 204 patients were divided into two groups based on their duration of illness: a short-term group with an illness duration of less than 5 years (*n* = 118) and a long-term group with an illness duration of 5 years or more (*n* = 86).

### Physical parameters

In addition to data collected from the medical records, a blood sample from each patient was collected on the first day or in the very early stage of therapy. Serum chemistry was performed in the laboratory of Kyushu University Hospital. Also, no patients took medicine to supplement electrolyte decrease until the blood tests were completed.

### Psychological parameters

All patients completed the Japanese versions of the Eating Disorder Inventory (EDI) [13] for the measurement of eating disorder pathology, the 20-item Toronto Alexithymia Scale (TAS-20) [14] for the assessment of alexithymia, the Parental Bonding Instrument (PBI) [15] for the assessment of their relationship with their parents, the State-Trait Anxiety Inventory (STAI) [16] to assess anxiety, and the Center for Epidemiologic Studies Depression Scale (CES-D) [17] to assess depression. All instruments were standardized and validated for use with Japanese patients [18–21]. All measures were completed during the first visit to the clinic.

### Data analysis

Differences between the two groups were analyzed using the Wilcoxon rank sum test after testing for a normal distribution with the Shaprio-Wilk test. The chi-square test was used to analyze categorical data. Stepwise multiple regression analyses were performed to identify predictors of duration of illness. All tests were considered statistically significant at *p* < 0.05. All statistical analyses were performed using JMP® version 14 for Mac OS (SAS Institute Inc., Cary NC, 1989–2019).

## Results

### Clinical characteristics

Table 1 shows the clinical characteristics for the study participants. A significantly higher rate of the restricting type of AN was observed in the short-term group(*p* = 0.0055). On the other hand, a significantly higher rate of the binge eating/purging type of AN was observed in the long-term group(*p* = 0.0010). Patients in the short-term versus the long-term group were significantly younger (19.40 ± 8.83 vs. 34.64 ± 10.32 years old; *p* < 0.0001, respectively) and their duration of illness was significantly shorter (1.77 ± 1.33 vs. 17.14 ± 8.86 years; *p* < 0.0001, respectively). Body mass index (BMI) did not differ between the two groups.

**Table 1:** Clinical characteristics of patients with AN

	Short	Long	*P* value
n	118	86	
Subtype (%)			
AN-R	68.64	25.58	0.0055
AN-BP	31.36	74.41	0.0010
Age	19.40±8.83	34.64±10.32	<0.0001
Duration of illness	1.77±1.33	17.14±8.86	<0.0001
BMI (kg/m^2^)	14.66±2.06	14.20±2.25	ns

*AN-R* Restricting anorexia nervosa, *AN-BP* Binge eating / purging anorexia nervosa, *BMI* Body mass index

### Somatic features

Table 2 shows the somatic features of the two groups. Total protein and serum albumin levels were significantly lower in the long-term group than in the short-term group (*p* = 0.0159 and *p* = 0.0014 respectively). In addition, statistically significant lower levels of potassium and chloride were observed in the long-term group (*p* = 0.0012 and *p* = 0.0002 respectively). Moreover, the serum calcium level was significantly lower in the long-term group than in the short-term group (*p* = 0.0009). No differences were observed for serum creatinine, blood urea nitrogen, or liver enzymes.

**Table 2:** Somatic features of patients with AN

	Reference value	Short	Long	*P* value
n		118	86	
TP (g/dl)	6.6 – 8.1	7.02±0.51	6.81±0.71	0.0159
Alb (g/dl)	4.1 – 5.1	4.68±0.46	4.24±0.60	0.0014
BUN (mg/dl)	8 – 20	14.12±4.98	14.99±12.49	ns
Cr (mg/dl)	0.46 – 0.79	0.63±0.14	0.70±0.34	ns
AST (U/l)	13 – 30	39.90±88.29	35.69±35.11	ns
ALT (U/l)	7 – 23	45.85±111.95	38.97±88.27	ns
Na (mmol/l)	138 – 145	140.23±2.93	138.96±3.93^)^	ns
K (mmol/l)	3.6 – 4.8	4.12±0.44	3.76±0.70	0.0012
Cl (mmol/l)	101 – 108	103.02±3.35	99.41±7.39	0.0002
Ca (mg/dl)	8.8 – 10.1	9.61±0.41	9.34±0.63	0.0009

*TP* Total protein, *Alb* Albumin, *BUN* Blood urea nitrogen, *Cr* Creatinine, *AST* Aspartate aminotransferase, *ALT* Alanine aminotransferase, *Na* Sodium, *K* Potassium, *Cl* Chloride, *Ca* Calcium

### Psychological features

Table 3 shows the psychological features of the two groups. The CES-D score (*p* = 0.0039) and both the state and trait scale scores from the STAI were significantly higher (*p* < 0.0001, *p* = 0.0011 respectively) in the long-term group than in the short-term group. The EDI total score of the long-term group was significantly higher than that of the short-term group (*p* < 0.0001). For the EDI subscales, significantly higher scores were observed in the long-term group for drive for thinness (*p* = 0.0020), lack of interoceptive awareness (*p* = 0.0070), bulimia (*p* < 0.0001), body dissatisfaction (*p* = 0.0010), ineffectiveness (*p* < 0.0001), perfectionism (*p* = 0.0367) and interpersonal distrust (*p* = 0.0304). The maturity fears subscale was the only one with no statistically significant difference between the two groups. The PBI total scores were not significantly different, but when examining the PBI subscales, the care scores were significantly lower (*p* < 0.0001) and the overprotection scores were significantly higher (*p* < 0.0001) in the long-term group. TAS-20 scores were relatively high, but not significantly different between the two groups.

**Table 3:** Psychological features of patients with AN

	Short	Long	*P* value
n	118	86	
	24.25±15.27	30.60±14.64	0.0039
STAI			
State	51.05±11.92	57.80±10.81	<0.0001
Trait	53.27±13.88	59.94±13.45	0.0011
EDI			
Drive for thinness	7.67±7.22	11.07±6.98	0.0020
Lack of interoceptive awareness	9.70±8.46	12.91±8.82	0.0070
Bulimia	4.46±6.60	10.18±8.14	<0.0001
Body dissatisfaction	12.38±6.44	15.37±5.99	0.0010
Ineffectiveness	11.42±7.77	16.23±7.50	<0.0001
Maturity fears	9.21±4.52	10.22±5.66	ns
Perfectionism	4.22±4.45	5.63±4.89	0.0367
Interpersonal distrust	8.38±4.64	9.86±4.74	0.0304
Total	67.37±37.20	89.38±40.50	<0.0001
PBI			
Care	27.63±7.64	21.96±9.64	<0.0001
Overprotection	10.66±7.93	15.56±8.78	<0.0001
Total	38.31±7.44	37.57±8.28	ns
TAS-20			
Difficulty identifying feeling	19.92±8.07	21.78±7.30	ns
Difficulty describing feeling	17.01±4.84	17.76±4.50	ns
Externally-oriented thinking	21.05±3.82	20.48±4.19	ns
Total	58.06±13.48	60.01±12.66	ns

*CES-D* The Center for Epidemiologic Depression Scale, *STAI* State-Trait Anxiety Scale, *EDI* Eating Disorder Inventory, *PBI* Parental Bonding Instrument, *TAS-20* Toronto Alexithymia Sclae-20

### Predictors of the duration of illness

Multiple regression analyses were performed to identify significant predictors of duration of illness (Table 4). Psychological variables that were mainly significant in the group analysis included the CES-D, STAI, EDI and its subscales, and PBI score. The PBI subscale of overprotection was found to be the only statistically significant predictor of duration of illness (β = 0.23, *p* = 0.0134).

**Table 4:** Predictors of duration of illness

Outcome	predictor	B	SE	β	t	*P*
Duration of illness	PBI overprotection	0.25	0.10	0.23	2.50	0.0134

*PBI* Parental bonding instrument

## Discussion

This study revealed that the clinical manifestations of AN differed according to the duration of illness. To our knowledge, this is the first study to demonstrate that parental bonding attitude is related to the chronicity of AN.

AN typically starts in adolescence with dietary restriction and becomes out of control [22]. Eddy et al. [23] found that 62% of patients with the restricting type of AN crossed over to binge eating/purging type of AN. This may be the reason why higher rates of the restricting type of AN were observed in the short-term group and higher rates of the binge eating/purging type of AN were observed in the long-term group in the current study.

Previous reports showed that recovery from AN became less likely for patients with a longer duration of illness, so early intervention was more likely to be effective [7, 8]. Steinhausen reported that the recovery rate appeared to be slower in patients who suffered from the illness for 10 years compared to patients with an illness duration of 4–10 years [24]. Moreover, family therapy was less effective for patients with early-onset AN that lasted for over 3 years [9]. Therefore, duration of illness is an important prognostic factor for patients with AN [1, 2]. The concept of patients with a severe and enduring eating disorder (SEED) has recently been emphasized, which has resulted in the characteristics of SEED being discussed worldwide [25–29]. SEED patients have been reported to have severe symptoms with a longer duration of illness, resulting in serious chronic physical complications [25]. However, a universal definition for SEED has not been fully established. Broomfield et al. [28] conducted a review of the literature on severe and enduring anorexia nervosa (SEAN) and found that the most common criteria for the definition of SEAN were duration of illness and previously failed treatment attempts. According to the literature review, when addressing the chronicity of AN, it is important to consider not only duration of illness but also treatment difficulty. Regarding duration of illness, a 7-year period of AN was most commonly used for defining SEAN, the second most commonly used was a 10-year period, and the third was a 5-year period. For the current study, we used a 5-year period of AN to divide the sample based on our clinical experience that a large proportion of patients with treatment difficulties had a duration of illness of 5 years or more. Few studies have examined differences in clinical features according to the duration of illness using both multiple physical and psychological parameters. In the current study, we could demonstrate that both the physical and psychological features of AN patients with illness duration of 5 years or more were more severe than those of AN patients with illness duration of less than 5 years. We also revealed that parental bonding style might be an important factor for the chronicity of AN. However, treatment response is also an important factor in SEED [30]. Research on longitudinal outcomes is needed to clarify SEED in the same sample.

Regarding the somatic features of patients with AN, total protein, serum albumin, serum chloride, and serum calcium levels were significantly lower among patients with a longer illness duration. The serum albumin level is commonly used as an indicator of nutritional status, but Narayanan et al. [31] reported that the albumin level did not correlate with weight status in AN. According to our results, BMI, which was extremely low in the study sample, did not differ between the two groups; however, serum albumin and total protein levels were significantly different between the groups. Huysentruyt et al. [32] examined serum albumin and pre-albumin levels of 75 adolescent patients with restricting type AN and found that none had a low serum albumin level, but 24% had a low pre-albumin levels. A normal total protein level was observed in the patients with AN whose BMI ranged from 7.9–16.8 kg/m^2^ [33]. Gaudiani et al. reported a higher prevalence of low albumin (66.7%) among AN patients aged > 40 years compared to patients aged < 30 years (6.5%) [34]. Therefore, serum albumin and total protein might be associated with aging and not reflect nutritional status, especially among young patients with AN who have a shorter duration of illness. However, the current study suggests that serum albumin and total protein might decrease over the longitudinal course of the illness.

Serum chloride and potassium levels are known to be associated with the frequency of purging behaviors, such as self-induced vomiting [35]. A prolonged or depressed QT interval in the electrocardiogram is often found in cases of hypokalemia [36]. The depressed QT interval and severe hypokalemia expose patients to a higher risk of sudden cardiac events, such as ventricular arrhythmia, which might be related to the sudden death of AN patients [37]. In the current study, serum potassium and serum chloride levels were significantly decreased in patients with a long-term duration of illness. This might be related to the higher proportion of patients with binge eating and purging in the long-term group. Although it is unclear whether alterations of potassium and chloride levels are related to the frequency of purging behavior or duration of illness, our results suggest that more careful monitoring of chloride and potassium levels is essential for patients with a longer duration of illness.

The serum calcium level of the long-term group was lower than that of the short-term group. Hypocalcemia is often found in patients with AN, which may result in chronic malnutrition or alkalosis [38]. On the other hand, Divasta et al. [39] reported that the serum calcium levels of 12 adolescent patients with AN were normal compared with those of age-matched healthy controls. Additionally, serum calcium is well-known to be associated with the serum albumin level. This may partly be affected by the decreased level of albumin of patients with long-term duration of illness.

All physical findings that were significant between the two groups were found to be negatively associated with duration of illness (data not shown); however, it is undeniable that the physical findings were affected by AN subtype and age.

Regarding the psychological features, depression and anxiety scores were significantly higher in the long-term group. A cross-sectional study revealed that depression and anxiety were related to eating disorder symptoms and anxiety was related to older age [40]. Thus, older age might have been a factor related to high anxiety in the current study. Moreover, for depressive symptoms, patients with a binge-purge subtype of AN or severe eating disorder pathology responded better to cognitive behavior therapy (CBT) for AN than to specialist supportive clinical management [41]. Therefore, especially among individuals with a longer duration of illness, having depressive symptoms or an binge eating / purging subtype might be important factors for choosing an appropriate therapy, such as CBT.

The EDI profiles of the two groups were clearly different, with the EDI total score being higher in the long-term group. This means that the eating disorder psychopathology of patients with a longer duration of AN was more severe than that of patients in the short-term group. Moreover, the subscale scores for of bulimia, body satisfaction, ineffectiveness, perfectionism, and interpersonal distrust were higher for the patients with longer duration of illness than for those in the short-term group. This suggests that eating disorder psychopathology may change over the course of AN. On the other hand, the subscale maturity fear was the only one where scores were not different between the two groups. This may mean that maturity fear exists through both earlier and later stages of the illness. Thus, our results might also indicate that illness duration of 5 years or more could lead to increased difficulty with therapy and lower likelihood of recovery. Therefore, early intervention is important for patients with AN. Enhanced CBT has been reported to be effective for the patients with severe and enduring anorexia nervosa whose duration of illness is more than 7 years [42]. This suggests that treatment for AN with longer duration of illness should not be given up because of their severe eating disorder psychopathology.

Maternal overprotection has been reported to be associated with AN [43]. Also, lower maternal and parental care bonding has been observed in patients with chronic AN [44]. In the current study, lower parental care and higher parental overprotection were observed in patients with longer duration of illness. Interestingly, among the psychological variables, the only predictor of longer duration of illness was parental overprotection. Although causality cannot be inferred from this study design, our findings suggest that parental overprotection is both a cause and a consequence of AN chronicity. Namely, parental overprotection may have existed before the onset of AN and may have influenced the onset of the eating disorder. Alternatively, prolonged eating disorder pathology might also increase parental overprotection. Therefore, parental overprotection might play an important role for the chronicity of AN, suggesting consideration of early family intervention as a prevention method.

Emotional disturbances have been reported in patients with AN [45, 46]. Alexithymia is a well-known emotional disturbance in AN patients, with high levels of alexithymia having been reported in previous research [47]. Bourke et al. [48] found that the prevalence of alexithymia in patients with AN was 77.1%, but alexithymia was unrelated to the amount of weight loss, duration of illness, severity of depression, and general psychoneurotic pathology. Several reports have indicated that alexithymia is correlated with depression and the EDI score [47, 49]. In a previous Japanese study, significantly higher TAS-20 scores were observed among younger patients with eating disorders who first visited a psychosomatic medicine clinic than among healthy controls [21]. Our data show that TAS-20 scores were high in both groups, which is consistent with previous reports identifying high rates of alexithymia among patients with AN. Treatments that focus more on emotional expression may be necessary for patients with AN.

Recently, a severity staging model for AN was proposed. The Clinician Administered Staging Instrument for AN (CASIAN) has been reported to be useful for developing stages of severity scores for weight / weight history, onset and illness duration, dietary control, compensatory behavior, and other psychological conditions, including depression [50, 51]. Taking these indicators into consideration, biopsychosocial treatment for AN should be required depending on the duration of illness and the degree of AN severity.

This study has several limitations. First, it is retrospective and cross-sectional; therefore, causality cannot be inferred. Future longitudinal studies are needed. Second, we did not consider the treatment status prior to the patients’ visit of our clinic. It is not deniable that this might in part affect our results. Third, duration of illness was associated with age, therefore, patient age might independently have influenced the findings. Additionally, because self-report measures were used in this study, patients may not always have provided honest answers because of the defensive nature of AN.

## Conclusions

The physical and psychological features of AN differ according to illness duration. Although the current study could not conclude that an illness duration of 5 years was a critical point between short-term and long-term illnesses, clinicians should be aware that AN in patients with an illness duration of at least 5 years could be clinically serious, requiring full physical and psychological assessment. Attention should also be given to the parental bonding styles that contribute to the disease chronicity and consider early family interventions.

## Data Availability

The datasets analyzed in the current study are available from the corresponding author upon reasonable request.

## Abbreviations

AN: Anorexia nervosa
BMI: Body mass index
CES-D: Center for Epidemiologic Studies Depression Scale
EDI: Eating Disorder Inventory
EOT: Externally oriented thinking
PBI: Parental Bonding Instrument
STAI: State-Trait Anxiety Inventory
TAS-20: 20-item Toronto Alexithymia Scale
